# Expression of genes and localization of enzymes involved in polyunsaturated fatty acid synthesis in rabbit testis and epididymis

**DOI:** 10.1038/s41598-022-06700-y

**Published:** 2022-02-16

**Authors:** Cesare Castellini, Simona Mattioli, Elena Moretti, Elisa Cotozzolo, Francesco Perini, Alessandro Dal Bosco, Cinzia Signorini, Daria Noto, Giuseppe Belmonte, Emiliano Lasagna, Gabriele Brecchia, Giulia Collodel

**Affiliations:** 1 Department of Agricultural, Environmental, and Food Science, University of Perugia, Borgo XX Giugno 74, 06123 Perugia, Italy; 2 Department of Molecular and Developmental Medicine, Policlinico Santa Maria alle Scotte, University of Siena, Viale Bracci 14, 53100 Siena, Italy; 3 Department of Veterinary Medicine, University of Milano, Via dell’Università, 6, 26900 Lodi, Italy

**Keywords:** Biochemistry, Cell biology, Molecular biology

## Abstract

The metabolism of polyunsaturated fatty acids (PUFAs) plays an important role in male reproduction. Linoleic and alpha-linolenic acids need to be provided in the diet and they are converted into long chain polyunsaturated fatty acids by steps of elongation and desaturation, exerted by elongases 2 (ELOVL2) and 5 (ELOVL5) and Δ5- (FADS1) and Δ6-desaturase (FADS2). This study aims to assess the gene expression and localization of enzymes involved in the synthesis of n-3 and n-6 long-chain PUFAs in control rabbits and those fed diets containing 10% extruded flaxseed. Enzyme and PUFA localization were assessed in the testes and epididymis by immunofluorescence. Testes showed high gene expression of FADS2, ELOVL2 and ELOVL5 and low expression of FADS1. Intermediate metabolites, enzymes and final products were differently found in Leydig, Sertoli and germinal cells. FADS2 was localized in interstitial cells and elongated spermatids; ELOVL5 in meiotic cells; FADS1 was evident in interstitial tissue, Sertoli cells and elongated spermatids; ELOVL2 in interstitial cells. Epididymal vesicles were positive for FADS1, ELOVL2 and ELOVL5 as well as docosahexaenoic, eicosapentaenoic, and arachidonic acids. This knowledge of fatty acids (FA) metabolism in spermatogenesis and the influence of diet on FA profile could help identify causes of male infertility, suggesting new personalized therapy.

## Introduction

Lipids are essential for spermatogenesis as they are crucial for the membrane remodelling of developing germ cells. The testes have a characteristic lipid composition with an amount of long-chain polyunsaturated fatty acids (LCP), predominantly docosapentaenoic acid (DPA, 22:5n-6) in rodents and docosahexaenoic acid (DHA, 22:6n-3) in rodents^1^ and other mammals^2,3^. Human and animals cannot synthesize n-6 or n-3 PUFA due to a lack of appropriate fatty acid desaturase and elongase enzymes (i.e. plants employ oleic acid to obtain linoleic acid, LA and alpha-linolenic acid, ALA using Δ12 and Δ15 desaturases) thus, they need dietary supply of LA (C18:2 n-6) and ALA (C18:3 n-3). LA and ALA are essential fatty acids, which need to be provided in the diet and they are converted into vital fatty acids (FAs, e.g. arachidonic [ARA C20:4n-6], eicosapentaenoic [EPA, C20:5n-3], n-3 DPA and DHA) by alternating steps of elongation and desaturation, exerted by elongases 2 (ELOVL2) and 5 (ELOVL5) and Δ5- (FADS1) and Δ6-desaturase (FADS2)^4^. LA and ALA and their metabolites, ARA, n-6 DPA, EPA, n-3 DPA and DHA, in reproductive tissues strongly influence the reproductive function^5–8^.

The mRNA levels of these key enzymes involved in FA metabolism have been investigated in the testis^9–13^; high mRNA levels of desaturase and elongase were detected in semen, indicating that alterations in FA synthesis may lead to male infertility.

Besides germ cells, different somatic cell types are present in the testis, including Leydig cells, myoid cells and Sertoli cells that constitute the microenvironment or the niche of the testis, which is essential for regulating normal spermatogenesis. Two distinct processes allow the accumulation of FAs in these cells, a passive diffusion through the lipid bilayer and/or protein-facilitated transport mediated by the glycoprotein CD36, which is widely expressed in Sertoli cells. At the same time, it is reported that Sertoli cells are more active in LCP metabolism than germ cells, which in turn are richer in polyunsaturated fatty acids (PUFAs)^14^. This correlates well with the high expression of Δ5- and Δ6-desaturase in Sertoli cells and with the low expression in germ cells^15^.

Consecutively, the epididymis modulates several sperm surface remodelling events and, in this regard, the role of PUFA metabolism may represent an interesting issue. During epididymal maturation, PUFAs remain almost stable^16^; however, the content of DHA is higher in testicular than in epididymal mouse sperm^17^. Recently, Gautier and co-workers described an active PUFA metabolism during spermatogenesis and epididymal sperm maturation in stallions^18^.

Moreover, the elongation/desaturation rate of PUFAs differs between species, and it is affected by sex, hormonal status and feed^8,19^. Because n-6 and n-3 FAs compete for the same enzyme pathways, their metabolism is largely affected by the availability of the ALA and LA substrates and by the affinity of these FAs for the different enzymes^20,21^. Accordingly, in some studies it was observed that dietary enrichment in n-3 and n-6 PUFAs increased the quality of fresh or post-thawing sperm of different animal species^22–25^.

In the present paper, characterization of the gene and enzyme expression involved in the synthesis of n-3 and n-6 LCP was done in rabbits. LCP biosynthetic metabolic pathway and PUFA localization were assessed by immunofluorescence in the testes and epididymis in rabbit bucks fed control or enriched (10% extruded flaxseed) diets, to better underline the role of ALA enrichment in the metabolism of the enzymes during spermatogenesis.

## Materials and methods

### Animals and experimental design

Ten New Zealand White rabbit bucks, 140 days old, were selected and divided into two experimental groups (n = 5 per group) (Table 1).

- Control (CNT) group was fed a standard diet ad libitum.
- Flax group (FLAX) was fed a standard diet supplemented with 10% extruded flaxseed.

**Table 1:** Formulation, proximate analysis and fatty acid profile of the control (CNT) and n-3 polyunsaturated fatty acid (PUFA)-enriched diet (FLAX).

Ingredients	Units	CNT	FLAX
Dehydrated alfalfa meal	g/kg	300	380
Soybean meal 44%	g/kg	150	100
Barley meal	g/kg	410	310
Wheat bran	g/kg	52	52
Soybean oil	g/kg	30	–
Extruded flaxseed	g/kg	–	100
Beet molasses	g/kg	20	10
Calcium carbonate	g/kg	7	7
Calcium diphosphate	g/kg	13.5	13.5
Salt	g/kg	7	7
DL-methionine	g/kg	0.5	0.5
Vitamin-mineral premix^a^	g/kg	10	10
Digestible energy (Mj/kg)*		10.8	10.6
Crude protein	g/kg	174	174
Ether extract	g/kg	47.7	47.2
Crude fibre	g/kg	122	137
Ash	g/kg	89	84
C16:0	g/kg	6.50	5.58
C16:1	g/kg	0.21	0.30
C18:0	g/kg	2.51	2.28
C18:1	g/kg	10.66	10.23
C18:2, LA	g/kg	17.12	15.17
C18:3 ALA	g/kg	9.23	12.08

*Estimated from De Blas and Mateos^26^.

^a^Per kg diet: vitamin A 11,000 IU; vitamin D3 2,000 IU; vitamin B1 2.5 mg; vitamin B2 4 mg; vitamin B6 1.25 mg; vitamin B12 0.01 mg; alpha-tocopheryl acetate 5 mg; biotin 0.06 mg; vitamin K 2.5 mg; niacin 15 mg; folic acid 0.30 mg; d-pantothenic acid 10 mg; choline 600 mg; Mn 60 mg; Fe 50 mg; Zn 15 mg; I 0.5 mg; Co 0.5 mg.—indicates that the specific ingredient was not included in the diet.

The experimental protocol involved 110 days of feeding. All methods are reported in accordance with ARRIVE guidelines for the reporting of animal experiments.

This study was conducted in accordance with the Guiding Principles in the Use of Animals and approved by the Animal Ethics Monitoring Committee of the University of Siena (CEL AOUS; authorization no. 265/2018-PR, ISOPRO 7DF19.23).

### Sampling of rabbit organs

At the end of the experiment, the rabbits were killed in the university facility after overdose of barbiturates as approved by Animal Ethics Monitoring Committee of the University of Siena. The testes and epididymis (both sides) were accurately removed, and portions were placed in sterile tubes, immediately snap-frozen using liquid nitrogen and stored at − 80 °C for evaluation of the gene (RT-PCR) and enzyme (immunohistochemistry) expression and FA profile by GC-FID. Five samples per organ were collected and analysed.

### Analytical determinations

#### Determination of gene expression in rabbit testis and epididymis

Total RNA from the testes was extracted from around 30 mg of frozen tissue using NucleoSpin RNA (Macherey‐Nagel, Germany) following the specific manufacturer protocol. RNA integrity was checked through electrophoresis in formaldehyde gel and the RNA concentration was determined with a NanoDrop 2000 spectrophotometer (Thermo Fisher Scientific, USA). An amount of 1 µg of total RNA was used to synthesize the cDNA using Superscript ii and Random Hexamers (Thermo Fisher Scientific) according to the manufacturer’s instructions. Primer-BLAST was used as a tool for primer design for the genes of interest: fatty acid desaturases (FADS1 and FADS2) and fatty acid elongases (ELOVL2 and ELOVL5) (Table S1)^27^. The relative gene expression levels were normalized to β2-microglobulin (β2-MG) and glyceraldehyde 3-phosphate dehydrogenase (GAPDH), as the housekeeping genes (Table S1).

The real-time PCR was conducted in triplicate for each biological sample, in a CFX96 real-time PCR Detection System (Bio-Rad, Hercules, CA, USA) using EvaGreen dye (Bio-Rad, Hercules, CA, USA). The optimized RT-PCR mixture consisted of total reaction volumes of 20 μL that contained 0.01 ng of cDNA, 10 μL of SsoFast EvaGreen Supermix (Bio-Rad, Hercules, CA, USA), 0.4 μM of each primer and sterile distilled water to reach the final volume. The PCR programme consisted of: 98 °C for 2 min, 40 cycles at 98 °C for 3 s and 60 °C for 10 s, 95 °C for 1 min, cooling at 70 °C for 1 min, and finally an increase to 95 °C at a 0.2 °C increase every 10 s, with measurement of fluorescence. Threshold cycles (Ct) were used to quantify the relative gene expression and normalized to the two above-mentioned housekeeping genes according to the ΔΔCt method^28^.

#### Determination of enzyme localization in rabbit testis and epididymis

The testes and epididymis of rabbit bucks fed control and n-3-enriched FLAX diets were cut into small blocks, treated with 10% buffered formalin for 24 h at 4 °C and then washed in water for 1 h. After fixation, the tissues were dehydrated in a graded ethanol series (50%, 75%, 95%, 100%) and cleared with xylene. The specimens were treated with three infiltrations of molten paraffin at 60 °C for 1 h and then they were allowed to solidify at room temperature. The obtained blocks were sectioned using a Leica RM2125 RTS microtome (Leica Biosystems, Germany); Sects. (4 µm) were collected on glass slides and stained by the haematoxylin–eosin method for routine histology. The paraffin sections from the testicular tissue of control and treated rabbits were deparaffinized with xylene, and then treated in a graded ethanol series (100%, 90%, 80%, 70%) for 5 min and, finally, in water to rehydrate the tissue. For antigen retrieval, the sections were washed and treated with heat-induced epitope retrieval 1 (HIER 1) buffer (10 mM sodium citrate) at pH 6 for 20 min at 95 °C. Specimens were treated overnight at 4 °C with the primary antibodies anti-FADS1 (Δ5-desaturase; Sigma-Aldrich, St. Louis, MO, USA) diluted 1 : 20, anti-FADS2 (Δ6-desaturase; Sigma-Aldrich, St. Louis, MO, USA) diluted 1 : 20, anti-ELOVL5 (elongase 5; Sigma-Aldrich, St. Louis, MO, USA) diluted 1 : 100, anti-ELOVL2 (elongase 2; Sigma-Aldrich, St. Louis, MO, USA) diluted 1 : 70, anti-DHA, anti-FITC-linked EPA and anti-PE-linked ARA (MyBioSource Inc., San Diego, CA, USA) diluted 1 : 50.

After three washes for 10 min in phosphate-buffered saline (PBS), the slides (excluding those treated with conjugated primary antibody) were incubated with goat anti-rabbit antibody Alexa Fluor® 488 conjugate (Invitrogen, Thermo Fisher Scientific, Carlsbad, CA, USA), diluted at 1 : 100, for 1 h at room temperature. The slides were washed three times with PBS and treated with 4′,6-diamidino-2-phenylindole (DAPI, Sigma-Aldrich, Milan, Italy) for 10 min, followed by washing with PBS for 10 min. Finally, the slides were mounted with 1,4-diazabicyclo[2.2.2]octane (DABCO, Sigma-Aldrich, Milan, Italy).

#### Determination of FA profile in rabbit diets, testis and epididymis

Lipids were extracted from the feed and different tissues according to Mattioli et al.^29^. To obtain fatty acid methyl esters, the lipid extract was dried with a rotary evaporator (Strike 10 Steroglass, Italy), and 1 ml of n-hexane was added. Finally, the trans-methylation procedure was performed with 0.5 ml of 2 M KOH–methanol solution at 60 °C for 15 min. To calculate the amount of each FA, heneicosanoic acid was used as the internal standard (C21:0, Sigma-Aldrich analytical standard). The recovery rate of the internal standard in the testis was 83% ± 3%.

The FA composition was determined using a Varian gas chromatograph (CP-3800) equipped with a flame ionization detector and a capillary column 100 m long × 0.25 mm × 0.2 μm film (WAX-10; Supelco, Bellefonte, PA, USA). Helium was used as the carrier gas with a flow of 0.6 mL/min. The split ratio was 1 : 20. The oven temperature was programmed as reported by Mattioli et al.^29^. Individual FA methyl esters (FAME) were identified by comparing the relative retention times of peaks in the sample with those of a standard mixture (FAME Mix Supelco, Sigma-Aldrich).

### Statistical analysis

All the numerical results (gene expression and FA profile) were analysed with a linear model analysing the effect of diet (control and flax)^30^. Results were expressed as LS means and differences were considered significant when *p* ≤ 0.05.

## Results

**Figure 1:**
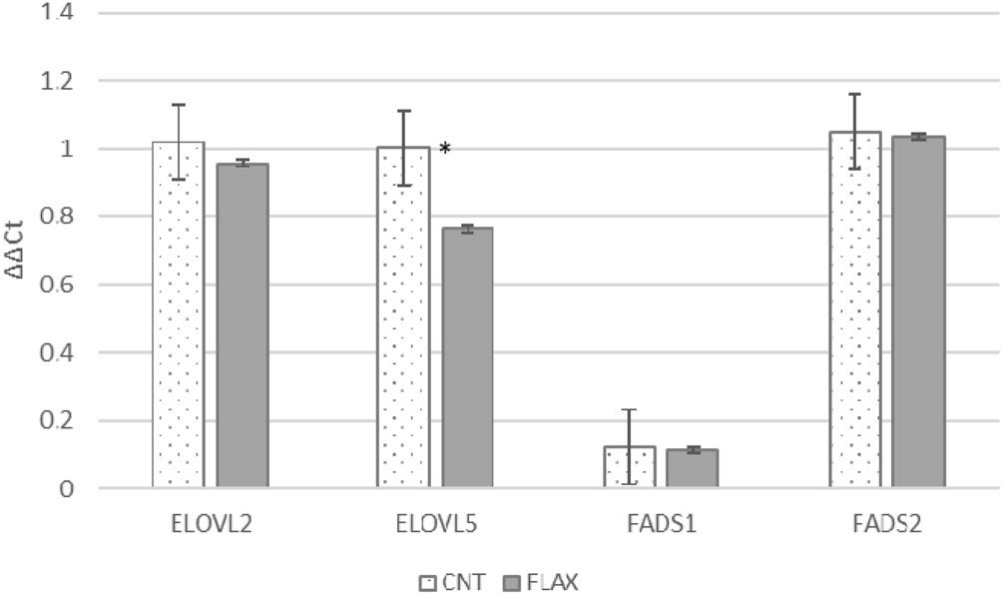
Expression of elongase 2 and 5 (ELOVL2 and ELOVL5) and Δ5- and Δ6-desaturase (FADS1 and FADS2) in rabbit testis. CNT: pointed bar; FLAX: full grey bar. *Significance of diet; comparison by t-test (*p* < 0.05).

The testes, independently of the diet administered, showed high expression of FADS2, ELOVL2 and ELOVL5 and lower expression of FADS1 (Fig. 1). FLAX administration partially affected the gene expression, testes showing significantly lower values only for the ELOVL5 gene.

To study the localization of the different enzymes, it is essential to understand their activity; we used immunofluorescence performed in rabbit testis and cauda epididymis tissues.

**Figure 2:**
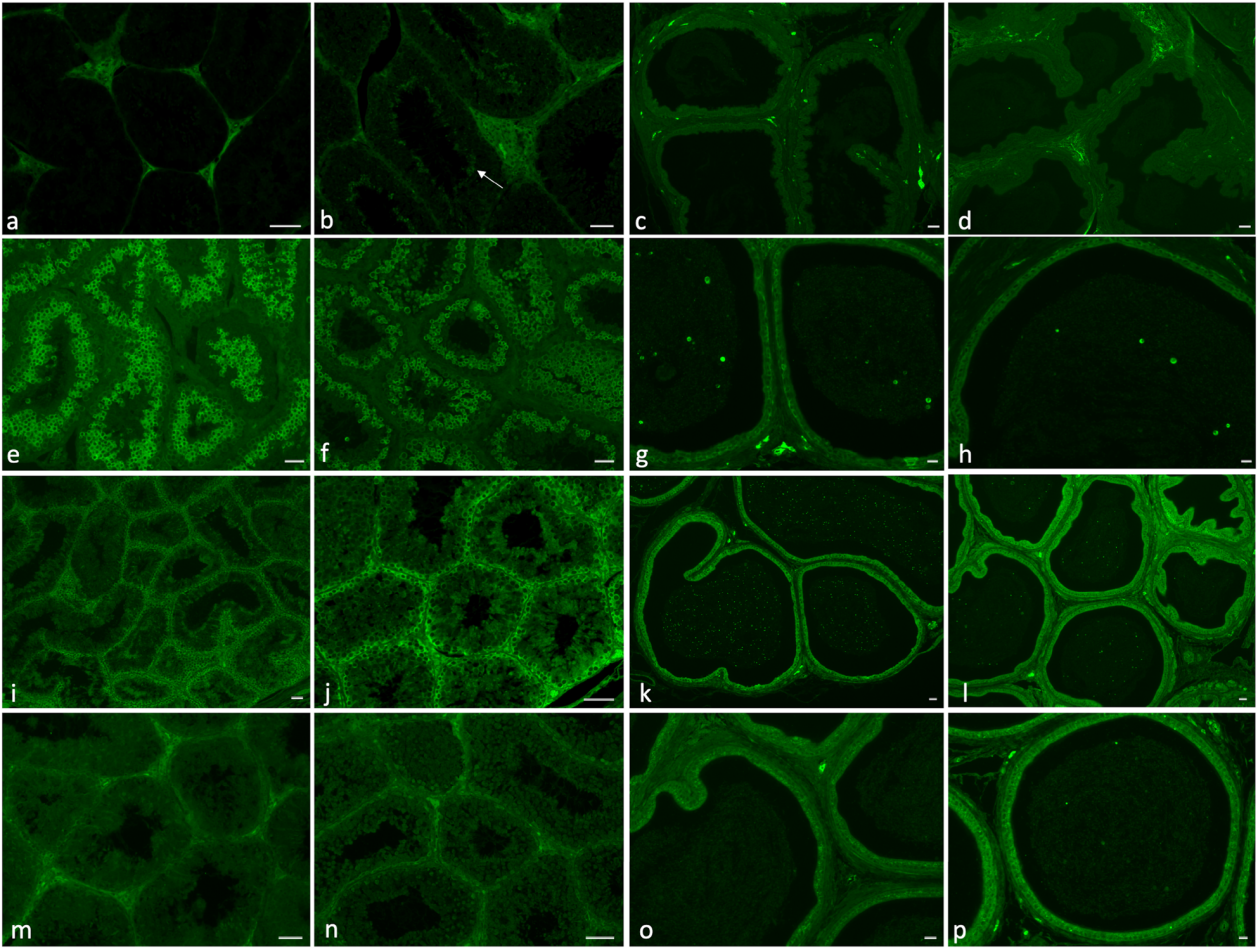
Immunolocalization of Δ6-desaturase (FADS2) in testis (**a**,**b**) and epididymis (**c**,**d**); very long-chain fatty acid 5 elongase (ELOVL5) in testis (**e**,**f**) and epididymis (**g**,**h**); Δ5-desaturase (FADS1) in testis (**i**,**j**) and epididymis (**k**,**l**); and very long-chain fatty acid 2 elongase (ELOVL2) in testis (**m**,**n**) and epididymis (**o**,**p**). A high labelling intensity was evident in the interstitial tissue in testis from rabbits fed control (**a**) and FLAX diets (**b**); in the latter, the FADS2 signal was present also in elongated spermatids (arrow). In the cauda epididymis (control **c**, FLAX **d**), FADS2 was absent from the principal cells; FLAX diet increased the presence of the enzyme in interstitial connective tissue. A high labelling intensity of ELOVL5 was shown in spermatocytes and round spermatids in testis from rabbits fed control (**e**) and FLAX diets (**f**). In cauda epididymis control and FLAX (**g**, **h** respectively), ELOVL5 appeared in principal epidydimal cells, in interstitial connective tissue and in numerous vesicles in the lumen where the spermatozoa were located. A clear signal of FADS1 was detected in Sertoli cells in testis from rabbits fed control (**i**) and FLAX diets (**j**); in the latter, the fluorescence intensity was increased and present in elongated spermatids. In the cauda epididymis, both control and FLAX (**k**, **l**), FADS1 was localized in interstitial connective tissue, basal and principal epididymal cells, and in a very large number of epididymal vesicles. A high labelling intensity of ELOVL2 was evident in the interstitial tissue in testis from rabbits fed control (**m**) and FLAX diets (**n**). In the cauda epididymis (**o**, **p**), ELOVL2 appeared localized in basal and principal epididymal cells as well as in interstitial connective tissue; a small number of vesicles appeared fluorescent in the lumen where the spermatozoa were located (more evident in FLAX diet). Bar: 50 µm.

In the testis of control rabbits, FADS2, acting on both LA and ALA, was clearly localized in interstitial cells (Fig. 2a); in the FLAX group, the fluorescence was also highlighted in the elongated spermatids (Fig. 2b).

In the cauda epididymis of both control and FLAX groups, FADS2 seemed to be present in connective epididymal tissue but it was not so evident in epididymal vesicles or in principal epididymal cells (Fig. 2c,d). In the epididymis of the FLAX group, the FADS2 signal increased in interstitial connective tissue.

ELOVL5 was mainly localized in meiotic cells (spermatocytes and round spermatids) and it was not present in spermatogonia, elongated spermatids, Sertoli cells or interstitial tissue (Fig. 2e,f). In the cauda epididymis, ELOVL5 was expressed in principal epididymal cells as well as in the epididymal vesicles (Fig. 2g,h). In addition, connective interstitial tissue showed the presence of the enzyme.

Δ5-Desaturase (FADS1) localization was evident in interstitial tissue, Sertoli cells and in spermatogonia (Fig. 2i); in the testis of rabbits fed FLAX diet, the signal was more evident and clearer also in the elongated spermatids (Fig. 2j).

In the cauda epididymis, both control and FLAX, FADS1 was localized in interstitial connective tissue, in basal and principal epididymal cells, and in a conspicuous number of epididymal vesicles (Fig. 2k,l).

ELOVL2 was localized in the interstitial cells of the testis (Fig. 2m,n), in interstitial epididymal connective tissue and in the principal and basal cells of the epididymis (Fig. 2o,p). The number of marked epididymal vesicles was higher in rabbits fed FLAX diet (Fig. 2p).

**Figure 3:**
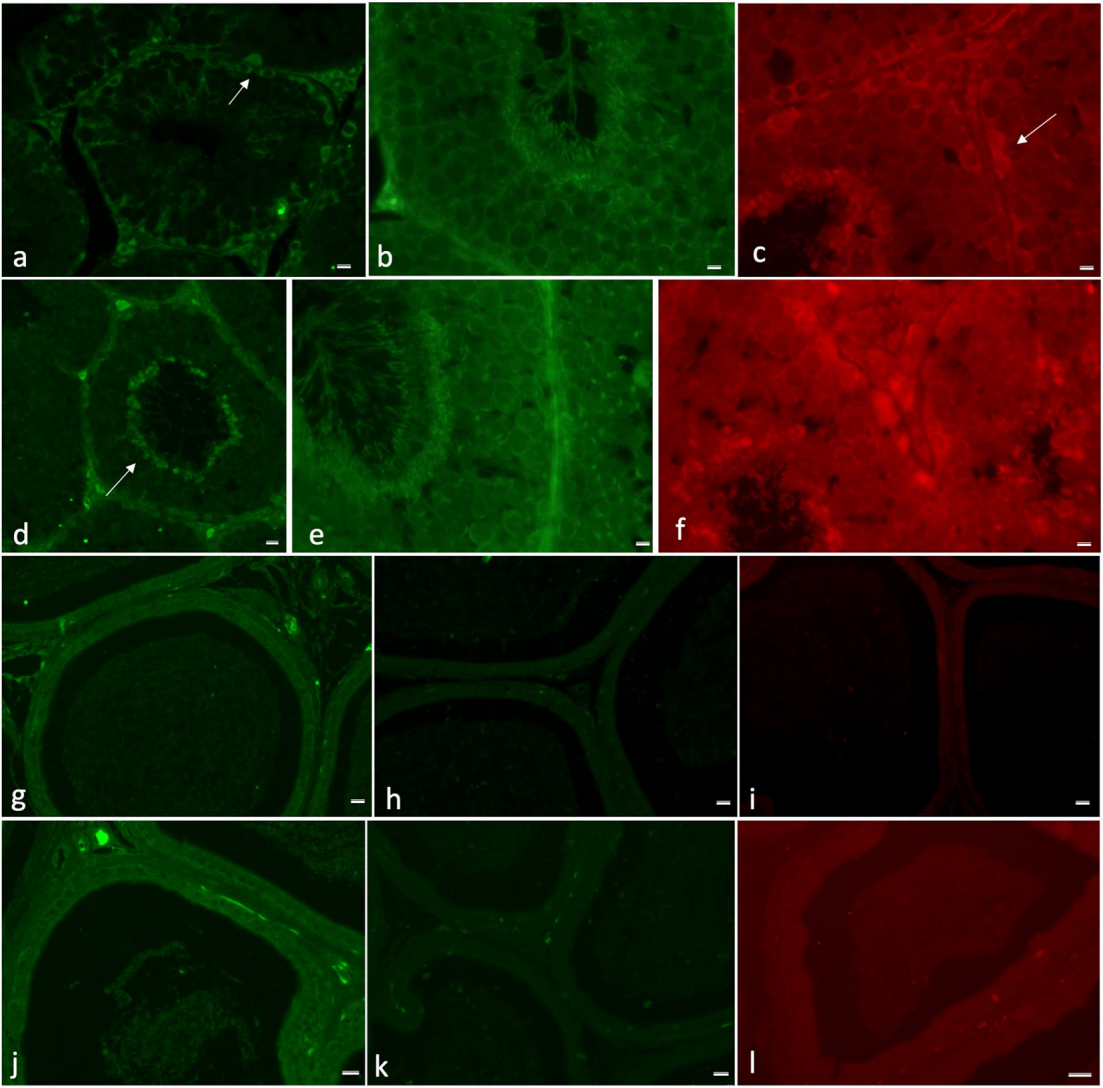
Immunolocalization of docosahexaenoic acid (DHA), eicosapentaenoic acid (EPA) and arachidonic acid (ARA) in testis and cauda epididymis of rabbits fed control and FLAX diets. In (**a**) a clear DHA localization was in interstitial tissue, Sertoli cells and spermatogonia; after FLAX diet (**d**), the signal was also evident in elongated spermatids (arrow); in (**b**) the EPA label appeared localized in the germ cells at different stages of maturation; after FLAX diet, the label was intense in the same cells (**e**); in (**c**) the ARA signal was detected in interstitial cells, Sertoli cells (arrow) and elongated spermatids of control testis. The same labelling was present in testis from rabbits fed FLAX diet (**f**). In the epididymis of rabbits fed control (**g**,**h**,**i**) and FLAX diets (**j**,**k**,**l**), in (**g**,**i**,**j** and **l)**, a limited number of vesicles in the lumen were labelled (DHA, **g** and **j**; ARA, **i** and **l**); on the contrary, in (**h**,**k)** a number of vesicles appeared labelled in the lumen where the spermatozoa were located (EPA). Bar: a–f, 10 µm, g–l, 50 µm.

To better understand the role of these enzymes, we also reported the localization of DHA, as well as EPA and ARA, in the testis, cauda epididymis and epididymal vesicles in rabbits fed control and FLAX diets (Fig. 3).

In the testis, DHA localization appeared in interstitial tissue, Sertoli cells and spermatogonia in control rabbits (Fig. 3a); after consumption of FLAX diet, it was also evident in elongated spermatids (Fig. 3d). The EPA label appeared in the germ cells (Fig. 3b) and it was intense after consumption of FLAX diet (Fig. 3e). The ARA signal was evident in interstitial tissue as well as in Sertoli cells and elongated spermatids of seminiferous tubules in both control (Fig. 3c) and FLAX diet groups (Fig. 3f).

In the epididymis from control and FLAX-fed rabbits, localization of the DHA label was detected (Fig. 3g,j) in interstitial connective tissue and principal cells as well as in a few vesicles. The signal appeared more evident in FLAX-fed rabbits (Fig. 3j). A reduced number of epididymal vesicles were also labelled using anti-ARA antibodies in the epididymis from control and FLAX-fed rabbits (Fig. 3i,l). In these last, the ARA localization appeared increased in interstitial connective tissue. Otherwise, many vesicles positive for EPA were detected (Fig. 3h,k) in epididymis from both control and FLAX-fed rabbits.

**Figure 4:**
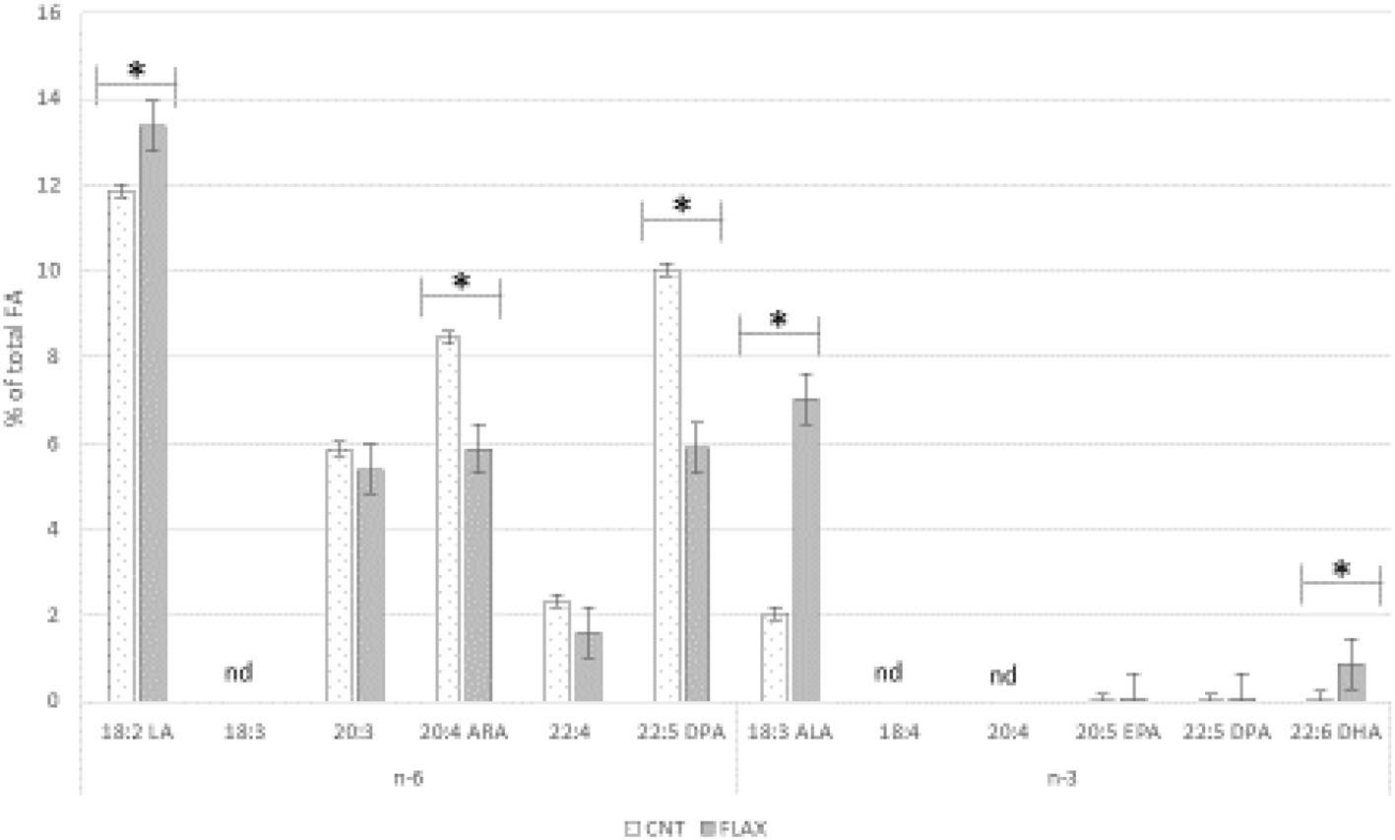
Testis PUFA profile of rabbit bucks fed control (CNT) or FLAX-enriched diet. LA: linoleic acid; ARA: arachidonic acid; DPA: docosapentaenoic acid; ALA: alpha-linolenic acid; EPA: eicosapentaenoic acid; DHA: docosahexaenoic acid. *Significance of diet; comparison by t-test (*p* < 0.05).

Figure 4 reports the PUFA profile of whole testis of control and FLAX groups. The PUFA profile showed significant differences in LA, ALA, 22:5n-6, ARA, and DHA, mainly in the group supplemented with flaxseed. LA, ALA and DHA increased in the FLAX group; ARA and 22:5n-6 were increased in the control group.

Other intermediate PUFAs (18:3 n-6, 18:4 n-3 and 20:4 n-3) are not evident in the PUFA profile, probably because they represented only metabolic steps, which will be easily converted into metabolites that were more relevant or were under the detection limits.

## Discussion

Apart from the liver, brain and adipocytes, the reproductive apparatus of mammals is widely involved in PUFA metabolism^25,31,32^. Indeed, mature sperm have a high level of LCP in their membranes, which assures fluidity and the movement of sperm with great speed^6,33^. This level of LCP is mainly guaranteed by liver metabolism^21,34^ which shows higher activity and expression of critical enzymes (i.e. FADS2, ELOVL2). However, several reproductive structures like the testes, epididymis and epididymosomes also exert a specific role^18,32^.

**Figure 5:**
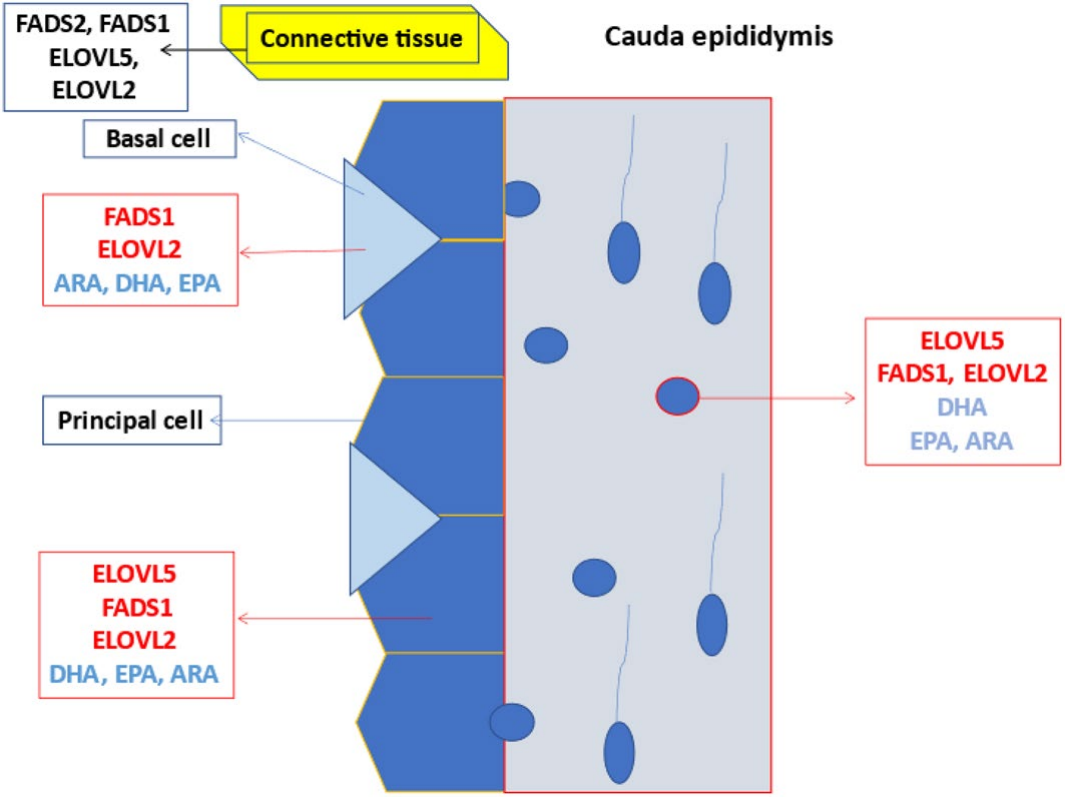
Schematic representation of enzyme and fatty acid localization in epididymal cells. ELOVL2: elongase 2; ELOVL5: elongase 5; FADS1: Δ5-desaturase; FADS2: Δ6-desaturase; DHA: docosahexaenoic acid; EPA: eicosapentaenoic acid; ARA: arachidonic acid. In the connective tissue of cauda epididymis all enzymes were expressed (FADS1 and 2, ELOVL2 and 5), while in the basal cell only FADS1 and ELOV2 were found and in the principal cell ELOVL5, 2 and FADS1. Conversely the FAs localization was similar (ARA, DHA and EPA in both cells). Furthermore, in the epididymal vesicles, the expression of ELOVL5,2 and FADS 1 was found.

In the present research, the gene expression of testis enzymes was slightly affected by dietary PUFAs (Fig. 5). Only the expression of ELOVL5 was downregulated when additional ALA was administered, probably since ELOVL5 is involved in the first steps of FA elongation, and FLAX diets provided a large amount of precursor (ALA)^35^. Meanwhile, no differences were found in the gene expression of ELOVL2, FADS2 or FADS1.

Similarly, in rat testis, no gene expression of any of the desaturase enzymes (stearoyl-CoA desaturase—SCD1, SCD2, FADS1 and FADS2) was induced by an increase in n-3 PUFA content^36^. However, in both the dietary regimes, gonads maintained a certain level of gene expression, confirming the role of reproductive tissues in LCP metabolism^29,32,37^.

In the liver, it is reported that many factors interfere with the gene expression of these enzymes, i.e. diet, body tissue and genetic variability^38^; nevertheless, supplementary dietary n-3 PUFAs reduce gene expression and the activity of SCD1, FADS1, FADS2 and ELOVL2^39,40^, whereas n-3 deficiency determines an increase of these enzymes^41^.

To our knowledge, a detailed analysis of the metabolic pathway of LCP in mammalian testis is shown here for the first time. The data show that the whole testis seems to be involved in LCP generation. Both n-6 and n-3 precursors (LA and ALA) were expressed, whereas intermediate metabolites and final products were differently found in the cells present in the testis (Leydig, Sertoli, germinal cells).

A certain amount of essential FAs reaches the testis^25^ through blood vessels, and different dietary administration of substrates (LA, ALA, EPA, DHA) may activate and modify this metabolism, inducing de novo synthesis.

Our study suggests that the testis is the preferential site of LA and ALA metabolism in the reproductive apparatus. Epididymal vesicles contain only a minimal amount of key enzymes of FA metabolism, e.g. FADS2. FADS2 is considered the rate-limiting step in LCP synthesis because it acts twice in this pathway, introducing a double bond to ALA and LA and to 22:5 both from n-6 and n-3 series, respectively^42–44^. This enzyme is widely evident in different cell lines of the testis (i.e. Leydig cells, elongated spermatids) and its products may be used at different developing cell stages.

A PUFA-enriched diet may influence Leydig cells and spermatogenesis^45^; our data seem to also suggest an intriguing role of the interstitial tissue. In fact, the increase of FADS2 in spermatids after consumption of FLAX diet could indicate that interstitial cells are able to support spermatogenesis in the production of metabolites, increasing at this stage also the production of EPA, ARA and DHA.

On the other hand, the fluorescent signal of FADS2 after dietary intake of FLAX increased in connective tissue but not in epididymal cells and vesicles, excluding their involvement in FADS2 activity.

This study also confirmed that ELOVL2 plays a crucial role in the lipid metabolic pathway, being required for the generation of very long-chain FAs (≥ 22 carbon atoms). ELOVL2 appears strongly localized in the testicular interstitial tissue and FLAX diet increases its quantity in both testis and epididymis. In the epididymis, the labelling is amplified in both connective and principal cells, determining the secretion of vesicles rich in this enzyme. FLAX diet also increases the presence of FADS1 and ELOVL5 in epididymal vesicles. Accordingly, the body of literature reports that both FADS2 and ELOVL2 knockout male mice are infertile since they cannot sustain sufficient levels of DHA, n-6 DPA and other PUFAs in the testis^35,46^.

Fish testis exhibits high expression of FADS1, FADS2 and ELOVL2 genes, which encode key enzymes to produce DHA. Recently, Bogevik et al.^7^ described phospholipid and LCP metabolism in Atlantic salmon (*Salmo salar*) testis during sexual maturation. ELOVL5, ELOVL2 and ELOVL4 mRNAs have also been detected in rat testis^5^ and were correlated with different maturation stages of sperm cells.

ELOVL5 metabolism takes place in the seminiferous tubules, in meiotic stages (spermatocytes and round spermatids), and probably uses as substrates the metabolites previously produced in Sertoli cells by FADS2; the resulting metabolic derivatives (20:3n-6 and 20:4n-3) represent substrates for FADS1 that was found in the stage of elongated spermatids. At the same time, the presence of ELOVL5 in epididymal vesicles suggests that vesicles may carry some preformed LCP (22:4n-6 and 22:5n-3) to the sperm membrane (see Fig. 5).

Sertoli cells have a fundamental role in normal and altered spermatogenesis. The numerous junctional complexes and membrane specializations made by Sertoli cells provide a scaffold and a peculiar environment for germ cell development^47^. The energy source of germinal cells originates from lactate produced by Sertoli cells, which in turn is provided mainly by mitochondrial oxidation of FAs.

**Figure 6:**
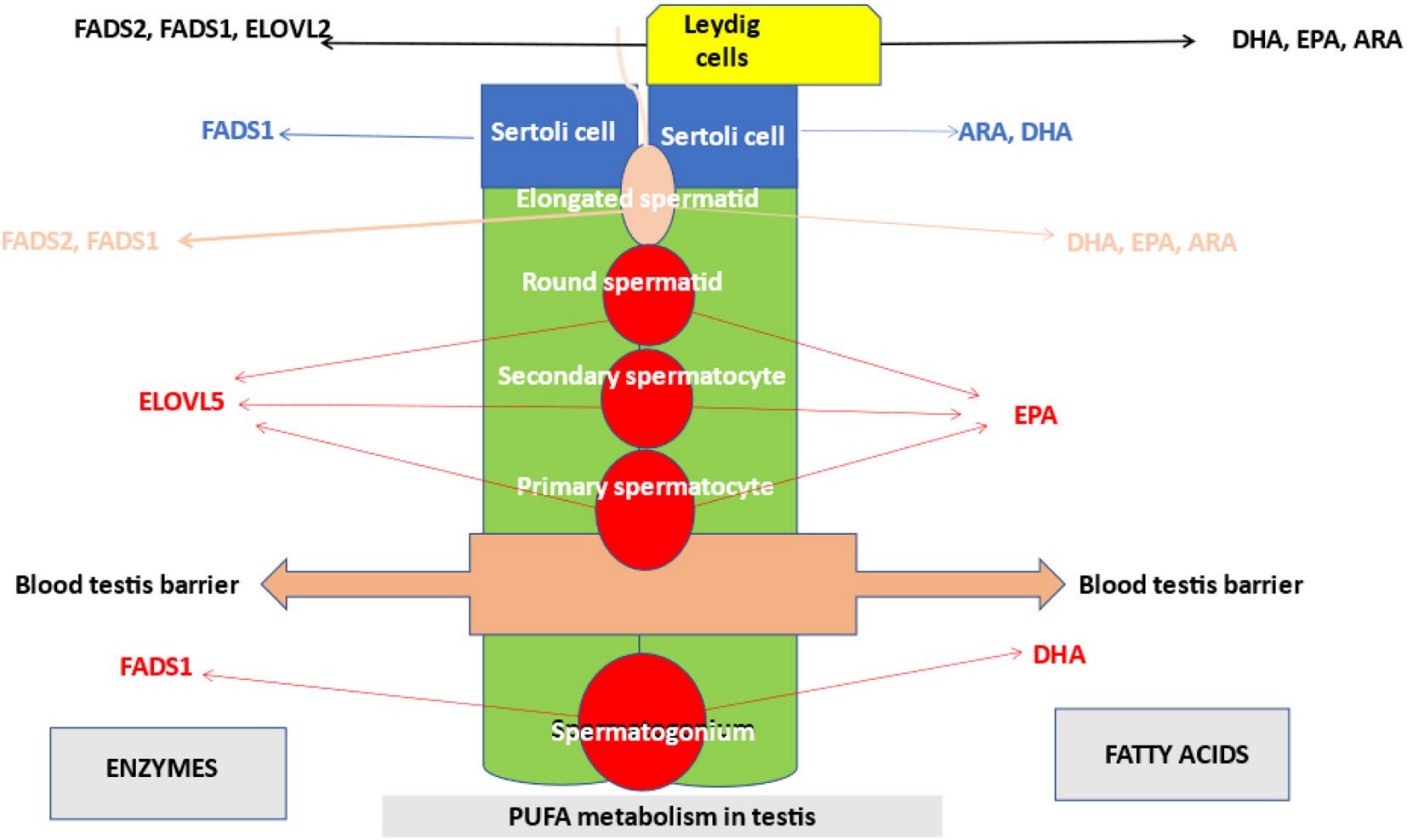
Schematic representation of enzyme and fatty acid localization in testis cells. The localization of the enzymes studied in germ, Sertoli and Leydig cells is shown on the left, whereas the localization of fatty acids is shown on the right. ELOVL2: elongase 2; ELOVL5: elongase 5; FADS1: Δ5-desaturase; FADS2: Δ6-desaturase; DHA: docosahexaenoic acid; EPA: eicosapentaenoic acid; ARA: arachidonic acid.

The metabolites of FADS1 activity (ARA and EPA) seem to have a role in Sertoli and Leydig cells and are present during spermatogenesis until the elongated spermatid stage. Production of the enzymes involved in the specific metabolic pathway increased in the presence of a diet enriched in ALA and became more evident in some stages of the cell germinal line, indicating that normal sperm maturation is dependent on these metabolites (Fig. 6). Luo et al.^48^ reported that a high-fat diet impairs spermatogenesis by regulating glucose and lipid metabolism in Sertoli cells.

Furthermore, the involvement of Leydig cells in PUFA synthesis could be also linked to the effect of LCP on sterol regulatory element binding protein (SREBP)^49^). However, the mechanisms by which these FAs regulate SREBP are not completely clear; probably EPA, DHA and ARA have far more capacity to inhibit SREBP processing than do the shorter-chain PUFAs (e.g. C18:1 n-9, LA and ALA)^49^. The immunolocalization of FADS1, FADS2 and ELOVL2 underlines the active metabolism of Leydig cells, where ARA and its metabolites influence cholesterol transport from the outer to the inner mitochondrial membrane to regulate steroidogenesis. In rats, ARA is secreted by Sertoli cells in an LH-dependent manner: LH recognizes the LHR of Leydig cells, which activates cAMP through G protein-coupled receptor (GPCR) signalling^50^. ARA can induce the release of Ca^2+^ from internal stores in round spermatids and pachytene spermatocytes; therefore, in the seminiferous tubule, unsaturated free FAs probably act as novel regulatory components of spermatogenesis^51^.

FADS1, FADS2 and ELOVL2 as well as ARA, EPA and DHA appeared widely localized in interstitial tissue. Matsuzaka et al.^52^ suggested that FADS1 and FADS2 expression is both regulated by transcription factors for FA metabolism and could be involved in lipogenic gene regulation by producing PUFAs. In general, interstitial cells take part in complex signalling interactions with both interstitial and tubular cell populations, influencing Sertoli cell function, spermatogenesis and immune regulation^53^.

In accordance with our data, in the stallion, Gautier et al.^18^, detected the presence of FADS1 in elongated spermatids and in epididymal cells even though, in their research, the localization of other enzymes was different (e.g. ELOVL5 was found in the interstitial compartment). Probably, each animal species has variability in enzyme expression, as demonstrated for the gene expression and enzyme activity^54^.

Moreover, the presence of enzymes in the epididymal vesicles suggests that vesicles can produce and add FAs to the sperm membrane with their fusion, as confirmed by the presence of DHA, EPA and ARA (Fig. 5).

It is known that epididymal vesicles, containing hundreds of proteins from different epididymal regions, have a role in establishing sperm competency in the complex process of reproduction^55^.

In this study, we clarify the main metabolic pathway to produce EPA, DHA and n-3 DPA that represent the most important PUFAs in rabbit testis and epididymis. A great support to understanding the steps of this process was comparison of the testis from control rabbits with that from those fed an ALA-enriched diet. In this group, the increased n-3 PUFA metabolism allowed us to better differentiate the enzyme localization and the different LCP during spermatogenesis.

The PUFA profile of the whole testis and the labelling of ARA, EPA and DHA are concordant with the enzymatic localization highlighted in this study. Testis showed a significantly higher proportion of n-6 PUFAs in control rabbits than in the FLAX group, where the most abundant FAs were ALA and DHA (*p* < 0.05). Moreover, the presence of these FAs in the epididymal vesicles suggests that they may be carriers able to modify the FA composition of sperm membrane.

This study carried out in rabbit testis may also represent a model for understanding the LCP mechanisms in humans. Different amounts of FAs have been reported in the sperm of fertile and infertile men^56,57^; in particular, ALA, EPA and DHA are reduced in oligoasthenoteratozoospermic patients^56,57^ and LA and ARA are low in fertile men. Indeed, it is known that the FA profile affects not only live cells and sperm motility but also capacitation, the acrosomal reaction and sperm–oocyte fusion, influencing male fertility^8^.

A deep understanding of physiological enzyme function could also help in characterizing some diseases, by emphasizing its role in the regulation of lipid storage and lipid oxidation in Sertoli cells^58^ and of testosterone in Leydig cells^59^.

Knowledge of the role of FA metabolism in sperm and spermatogenesis, and the influence of dietary FAs on the sperm FA profile, could help in identifying potential causes of male infertility, suggesting new personalized therapy.

## Supplementary Information

Supplementary Information.
