# Supplementary material for: Expression of genes and localization of enzymes involved in polyunsaturated fatty acid synthesis in rabbit testis and epididymis
Source: Sci Rep. 2022 Feb 16;12:2637. doi: 10.1038/s41598-022-06700-y (PMC8850619; doi:10.1038/s41598-022-06700-y)
Supplement: Supplementary file 1 — Supplementary Information. [file 41598_2022_6700_MOESM1_ESM.docx]

**Supplementary materials**

**Table S1.**  Rabbit primers used for RT-PCR

| **GENE** | **Product size (bp)** | **Primers** |
| --- | --- | --- |
| FADS1 | 74 | F:  TCTGCAGTGCTGCTCAGTGT  R: AAGACGGACAGGTGCCCAAA |
| FADS2 | 123 | F: ATCCCTTTCTACGGCATCCT  R: GGGTTCAAGGTCAATCTCCA |
| ELOVL2 | 92 | F: GGCTGGGCAACAAATCCATG  R: CGCGGAGAGAAGAGTGATCC |
| ELOVL5 | 159 | F: TTCTCTTGCCGGGGCATTTT  R: GACACGGATAATCTTCATGTCCG |
| β_2_-MG | 126 | F: GTCCACACTGAATTCACGCC  R: CTCAGACCTCCATGCTGTTGA |
| GAPDH | 53 | F: GTCGGAGTGAACGGATTTGG  R: AAAGCAGCCCTGGTGACCA |
